# The Relationship Between Income Inequality and the Palliative Function of Meritocracy Belief: The Micro- and the Macro-Levels Both Count

**DOI:** 10.3389/fpsyg.2021.709080

**Published:** 2021-10-08

**Authors:** Márton Hadarics, Anna Kende, Zsolt Péter Szabó

**Affiliations:** ^1^Department of Social Psychology, ELTE Eötvös Loránd University, Budapest, Hungary; ^2^Department of Ergonomics and Psychology, Budapest University of Technology and Economics, Budapest, Hungary

**Keywords:** palliative ideology, subjective well-being, meritocracy belief, social inequality, system justification

## Abstract

In the current paper, we report the analysis of the relationship between meritocracy belief and subjective well-being using two large international databases, the *European Social Survey Program* (*N* = 44,387) and the *European Values Study Program* (*N* = 51,752), involving data gathered from 36 countries in total. We investigated whether low status individuals are more likely to psychologically benefit from endorsing meritocratic beliefs, and the same benefits are more pronounced in more unequal societies. Since meritocracy belief can function as a justification for income differences, we assumed that the harsher the objective reality is, the higher level of subjective well-being can be maintained by justifying this harsh reality. Therefore, we hypothesized that the palliative function of meritocracy belief is stronger for both low social status (low income) individuals, and for those living in an unequal social environment (in countries with larger income differences). Our multilevel models showed a positive relationship between meritocracy belief and subjective well-being, which relationship was moderated by both individual-level income status and country-level income differences in both studies. Based on these results, we concluded that the emotional payoff of justifying income inequalities is larger if one is more strongly affected by these inequalities.

## Introduction

According to Oxfam International (2016)—an international charity organization—the richest 1% of the world owns more material resources than the bottom 99%. Income inequalities have been continuously growing in countries with the largest populations, like China, India, the US and Indonesia (Hasell, 2018), and a similar trend can be found in most European countries (Blanchet et al., 2019). It seems that social movements, or traditional political parties cannot put a stop to this tendency, suggesting that income inequalities do not have the mobilizing power that would curb this trend. The greatest psychological paradox of social inequalities is that not just the people on top of the social hierarchies, but also at the bottom are motivated to endorse ideologies that justify unequal social arrangements and depict them as legitimate (for the theory of system justification see Jost and Banaji, 1994; Jost, 2020; for the theory of social dominance explaining the endorsement of social hierarchies from an evolutionary perspective see Sidanius and Pratto, 1999). Specifically, material inequality can be legitimized by belief in meritocracy, in social mobility, and protestant work ethic, all of which suggest that people on top deserve their high, and people at the bottom deserve their low status (Jost and Hunyady, 2005).

Apart from preserving the hierarchy itself, these ideologies also contribute to people’s well-being. Acceptance of ideological beliefs that frame inequality as legitimate can temporarily protect one’s subjective well-being and serve as a buffer against negative emotions that may stem from the realization of both unearned privileges on the top of the social hierarchy and structural obstacles to equal opportunities at the bottom of the hierarchy (Jost and Hunyady, 2003). Although this buffer function might also depend on the actual extent of inequalities and one’s own position within the hierarchy (e.g., Napier and Jost, 2008; Osborne and Sibley, 2013; Sengupta et al., 2017), we know little about the differences in the palliative effects within and across social contexts with different degrees of inequality. Using multinational surveys, we examine the consistency of the link between subjective well-being and meritocracy belief, one of the most important legitimizing ideologies of income inequality. We also test how societal level of income inequalities and personal income status influence this relationship.

### Meritocracy Belief as a Legitimizing Ideology

The palliative function of justifying ideologies has been tested in connection with beliefs, like conservatism (Napier and Jost, 2008), symbolic prejudice (Sengupta et al., 2017), or general system justification (Vargas-Salfate et al., 2018), none of which is specifically targeting the problem of income inequalities. However, we expect the largest palliative effect of ideological beliefs if the specific hierarchical arrangements corresponding with the justifying ideologies, as it is suggested in the system-justification literature (e.g., Sengupta et al., 2015; Bahamondes-Correa et al., 2019). Accordingly, in terms of income status and income inequality, the palliative effect may be the strongest in case of an ideological belief that specifically justifies income inequalities, such as the belief in meritocracy.

Meritocracy, and specifically income meritocracy refers to the idea that income differences reflect (in a descriptive sense) or should reflect (in a prescriptive sense) real differences in talent and effort. In a normative sense, meritocracy is a popular idea, since it reflects the distributional principle of equity (Deutsch, 1975). In a descriptive sense, it also has a legitimizing function, since it frames income differences as just and well-deserved (Kluegel and Smith, 1986; Jost and Hunyady, 2005). In this way, descriptive meritocracy belief offers an explanation for different groups’ and individuals’ social status and wealth, which justifies and consequently contributes to maintaining the *status quo*. Accordingly, research shows that those endorsing descriptive meritocracy belief tend to blame low-status groups for their unfavorable situation, explain their low status with negative internal characteristics of the group, and disapprove affirmative policies targeting low-status groups (e.g., Fraser and Kick, 2000; Son Hing et al., 2002; Levy et al., 2010; Madeira et al., 2019). Furthermore, the results of Zimmerman and Reyna (2013) indicate that even prescriptive meritocracy can have a system justifying aspect, since in their study, high-status (vs. low-status) participants were more motivated to endorse prescriptive meritocracy in order to legitimize the *status quo*.

The relationship between meritocracy belief and accepting the *status quo* can be observed among members of low-status groups too. Those with strong descriptive meritocracy belief tend to accept their own disadvantaged position, perceive it as fair and deserved, and engage in negative auto-stereotyping (McCoy and Major, 2007; Rüsch et al., 2010; Wiederkehr et al., 2015). Meritocracy belief is also related to personal views on macro-level income distribution. Those perceiving society as not meritocratic tend to prefer more equal income distribution, while those with a stronger descriptive meritocracy belief tend to agree with high levels of income inequality (Mitchell and Tetlock, 2009; Larsen, 2016). In short, meritocracy belief, along with other ideological elements like belief in social mobility or protestant work ethic, is an essential ideological tool for justifying economic inequalities (Jost and Hunyady, 2005).

### The Palliative Effects of Justifying Ideologies

Income differences take a toll on the well-being of individuals both on a micro- and a macro-level, that is, both country-level income inequality and low individual income are related to different indicators of psychological well-being (e.g., Diener et al., 1995; Diener and Oishi, 2000; Boyce et al., 2010; Oishi et al., 2011; but see Berg and Veenhoven, 2010; Zagorski et al., 2014). However, people employ various strategies to cope with status and resource inequalities by endorsing different ideological beliefs as justification and rationalization. It means that endorsing beliefs and ideologies that describe disadvantage of social groups as either fair or natural, or deny the disadvantage, can temporarily boost the subjective well-being of a person (Jost and Hunyady, 2003, 2005). This seems to be a prevalent strategy because of its psychological benefit, like a palliative function. In other words, the justification of an unequal *status quo* has a direct payoff in terms of short-term subjective well-being.

This temporary palliative effect has been revealed in connection with a number of ideological beliefs. One common point in these beliefs is that they legitimize inequalities either in general or in terms of specific hierarchical arrangements like gender, racial, or economic inequalities. Accordingly, research shows that subjective well-being correlates with the endorsement of conservatism (Napier and Jost, 2008; Onraet et al., 2013; Butz et al., 2017), benevolent and hostile sexism (Napier et al., 2010; Hammond and Sibley, 2011; Connelly and Heesacker, 2012; Vargas-Salfate, 2017), symbolic racism (Sengupta et al., 2017), general and group-specific system justification (Rankin et al., 2009; Osborne and Sibley, 2013; Vargas-Salfate et al., 2018; Bahamondes-Correa et al., 2019), or the direct denial of the disadvantageous position itself (Bahamondes-Correa et al., 2019; Suppes et al., 2019). Most importantly related to our topic, there is also empirical evidence showing that subjective well-being is related to descriptive meritocracy belief, what is explained by the palliative effect of the latter (Napier and Jost, 2008; McCoy et al., 2013). Furthermore, Kelley and Evans (2017) report a positive correlation between prescriptive meritocracy and subjective well-being, what brings up the possibility that this latter form of meritocracy belief can have a palliative function too.

Some studies suggest that the palliative effect is stronger for low than high-status groups. This is based on the idea that the justification of inequalities helps people cope with harsh realities, and since these realities are harsher to low status-groups, they can psychologically benefit more from justifying it. In accordance with this idea, Osborne and Sibley (2013) found that general system justification reduced psychological distress for New Zealanders, and this effect was stronger when perceived individual deprivation was high (vs. low). Another study showed that ethnic-specific system justification mitigated the well-being gap between ethnic minority and majority members, and similarly, gender-specific system justification enhanced subjective well-being to a larger extent for women than for men (Bahamondes-Correa et al., 2019). Furthermore, it was found that the palliative function of general system justification was stronger for those with a low (vs. high) social status in China (Li et al., 2020).

However, other studies suggest the opposite association and argue that the palliative effect might be weaker for low-status groups. Although legitimizing ideologies can rationalize the underdog position, it can also undermine self-esteem and well-being by framing their low-status as deserved, just, or natural (O’Brien and Major, 2005; Rankin et al., 2009). These ideas are supported by longitudinal results showing that although the palliative effect can be observed only in the short run, however, after a while the positive relationship between subjective well-being and legitimizing the *status quo* turns into a negative one for low-status groups (Harding and Sibley, 2013; Godfrey et al., 2019).

Finally, no such differentiated effects were identified by others, suggesting that system-justifying ideologies promote the well-being of both high- and low-status group members equally. For example, Sengupta et al. (2017) found that symbolic racism showed a positive relationship with New Zealanders psychological well-being regardless of their ethnicity. A multinational study conducted in 18 countries found that general system justification correlated with multiple indicators of psychological health and well-being, and these relationships were not affected by subjective social status (Vargas-Salfate et al., 2018). Similarly, both Napier et al. (2010) and Connelly and Heesacker (2012) revealed a positive relationship between subjective well-being and the acceptance of sexism but did not find a difference between men and women in terms of the strength of this palliative effect.

Contextual factors can also moderate the palliative effect of system-justifying ideologies. In most cases these moderations show that the harsher the reality is, the higher level of well-being can be achieved by justifying or denying that reality. For example, by analyzing European Social Survey data Hadarics (2021) found that climate change denial has a stronger palliative effect in countries that are more exposed to the detrimental effects of climate change. Based on World Values Survey data, Onraet et al. (2017) confirmed that the relationship between subjective well-being and right-wing ideologies is stronger in countries with higher levels of social threat.

System-justification has larger payoff in terms of well-being when inequality is larger in a given context too. When status differences are larger people can be more motivated to justify these differences, and this justification can boost their well-being to a larger extent compared to situations where social inequalities are more moderate. Thus, system-justification has larger payoff in terms of subjective well-being when inequality is larger. Based on longitudinal data from the General Social Survey from the United States Napier and Jost (2008) found that in times of larger income inequalities, conservative (vs. liberal) political preferences served as a protective factor against unhappiness. Sengupta et al. (2017) revealed that the relationship between symbolic racism and subjective well-being was stronger in areas of New Zealand with high (vs. low) income differences (a proxy for supposed regional ethnic inequalities).

Furthermore, the results of an international study involving data from 32 countries from the World Values Survey showed that hostile sexism (without benevolent sexism counterbalancing it) predicted high subjective well-being in countries with a low level of gender equality, but the same relationship was negative in more equal countries (Napier et al., 2010). On the other hand, country-level income inequalities did not moderate the relationship between general system justification and subjective well-being in a longitudinal study of 18 countries (Vargas-Salfate et al., 2018).

### Research Question and Hypotheses

Although the positive relationship between meritocracy belief and subjective well-being has been identified in some studies (Napier and Jost, 2008; McCoy et al., 2013), according to our knowledge, the palliative effect of (income) meritocracy belief has not been directly tested in connection with the level of income inequality and one’s personal income status. We hypothesized a connection between income inequality and the temporary palliative effect of meritocracy belief. As we have seen, the palliative effect of system-justification can be stronger for members of low-status groups (Osborne and Sibley, 2013; Bahamondes-Correa et al., 2019). Therefore, we hypothesized that the poor can benefit more from endorsing meritocracy belief in term of their subjective well-being compared to the rich (H1). Furthermore, when social inequalities are large, people are more motivated to justify inequalities, therefore, endorsing different justifying beliefs can have a stronger palliative effect in a more unequal environment (Napier and Jost, 2008; Napier et al., 2010; Sengupta et al., 2017). Based on this we expect that accepting meritocracy belief is especially effective for preserving the subjective well-being in an environment where income distribution is highly unequal (H2).^1^

## Materials and Methods

To test the assumptions about personal and contextual income inequalities described above, we relied on two large-scale international survey databases: the European Social Survey (ESS) and the European Values Study (EVS). As it was presented above, such large-scale multinational survey datasets have already been applied successfully to test the moderating effect of country-level contextual factors in the palliative function of ideological thinking (Napier and Jost, 2008; Napier et al., 2010; Onraet et al., 2017; Hadarics, 2021). Both the ESS and the EVS are academically driven international survey programs that have been administered regularly with the participation of more than 30 European countries in both cases. These surveys measure the attitudes, beliefs, and behavior patterns of diverse populations across a wide range of European countries, which makes them an ideal tool for international comparative studies. In the case of the ESS, we analyzed the dataset from the 8th data collection round (European Social Survey Round 8 Data, 2016). This database contains data collected from probabilistic representative samples of 23 countries. In the case of the EVS, we relied on the dataset from the most recent (5th) data collection round (European Values Study, 2020), which contains data collected from probabilistic representative samples of 34 countries.

### Measures From the European Social Survey

Subjective well-being: For the assessment of psychological well-being we selected the self-reported happiness and life satisfaction items. In the ESS core questionnaire, respondents indicate both their personal level of happiness and life satisfaction by two items on an 11-point scale (happiness: *“Taking all things together, how happy would you say you are?”*; 0 = *Extremely unhappy*, 10 = *Extremely happy*; life satisfaction: *“All things considered, how satisfied are you with your life as a whole nowadays?”*; 0 = *Extremely dissatisfied*; 10 = *Extremely satisfied*). Before the analysis, we merged these variables into a combined psychological well-being score, what was enabled by the substantial correlation between the two items in each country (*r*_*mean*_ = 0.69; *r*_*range*_ = 0.57–0.76).

Meritocracy belief: The item measuring *‘belief about the positive functioning of a meritocratic reward system’*^2^ was used as an indicator for the acceptance of (income) meritocracy belief: *“Large differences in people’s incomes are acceptable to properly reward differences in talents and efforts.”* (1 = *Agree strongly*; 5 = *Disagree strongly*). The scores of this scale were reversed so that higher numbers indicated higher level of agreement. Based on its wording, the applied ESS item can be interpreted both in a descriptive and a prescriptive sense, depending on whether respondents consider *“large differences in people’s incomes”* as actual or hypothetical ones.

Income status: Respondents indicated the income of their households on a 10-point scale. This 10-degree scale was developed individually for each country within the ESS project, following the same methodology. Each degree of the scale means one decile of income distribution on the basis of the median income as a point of reference.^3^

Income inequality: National income inequality was measured by the Gini coefficient (Yitzhaki, 1979) for each country. We applied the Gini score for every country from the year in which the data was collected in that particular country (either 2016 or 2017). Data for this variable was obtained from the relevant Eurostat (2020) and The World Bank (2020) databases.

Control variables: We included control variables in the analyses both on individual and national levels. These included respondents’ gender (0 = Man; 1 = Woman), age, education (according to the levels of the International Standard Classification of Education), subjective health condition (1=very bad; 5=very good), self-reported religiousness (0=not at all religious; 10=very religious), and left-right ideological identification (0=left; 10=right). This latter two variables were important to be included because of the well documented happiness gap between conservatives and liberals (Napier and Jost, 2008; Onraet et al., 2013) on the one hand, and religious and non-religious people on the other (Poloma and Pendleton, 1990; Diener and Ryan, 2009) also because of the relationship between meritocratic beliefs and right-wing conservatism (e.g., Napier and Jost, 2008; Son Hing et al., 2011). On the national level, we controlled for the quality of life in each country indicated by the Human Development Index (HDI), which is a global measure developed within the United Nations Development Programme (2020) composed by measures of income, age expectancy, and educational level. We applied the HDI for every country from the year in which the data was collected in that particular country (either 2016 or 2017).

### Measures From the European Values Study

Subjective well-being: Again, for the assessment of psychological well-being, we selected the self-reported happiness and life satisfaction items of the questionnaire. In EVS, respondents indicate their personal level of happiness on a 4-point scale, and their life-satisfaction on a 10-point scale (happiness: *“Taking all things together, would you say you are:”*; 1 = *very happy*, 4 = *not at all happy*; life satisfaction: *“All things considered, how satisfied are you with your life as a whole these days?”*; 1 = *dissatisfied*; 10 = *satisfied*). Before the analysis, we merged these variables into a combined psychological well-being score after transforming the scores to common 11-point scale. This was enabled by the substantial correlation between the two items in each country (*r*_*mean*_ = 0.51; *r*_*range*_ = 0.42–0.61).

Meritocracy belief: A bipolar item was used as the indicator of the acceptance of (income) meritocracy belief using a 10-point scale (1 = *“Incomes should be made more equal.”*; 10 = *“There should be greater incentives for individual effort.”*). This item sets prescriptive income egalitarianism against a preference for a meritocratic rewarding system, where, consequently, a higher score would indicate a higher level of preference for meritocracy, where material rewards are in accordance with individual efforts. Since an answer to this item indicates a personal preference, the item can be considered as an indicator of prescriptive meritocracy belief.

Income status: Respondents indicated the income of their household on a 10-piont scale. This 10-degree scale was developed individually for each country within the EVS project, following the same methodology as in the ESS. Each degree of the scale means one decile of income distribution on the basis of the median income as a point of reference.^4^

Income inequality: National-level income inequality was indicated by the Gini coefficient for each country. We applied the Gini score for every country from the year in which the data was collected in that particular country (2017, 2018, or 2019). The data for this variable was obtained from the relevant Eurostat (2020) and The World Bank (2020) databases.

Control variables: We included control variables both on individual and national levels. These included respondents’ gender (0 = Man; 1 = Woman), age, education (according to the levels of the International Standard Classification of Education), subjective health condition (1 = very poor; 5 = very good), self-reported religiousness (*“Do you belong to a religious denomination?”*; 0 = *no*; 1 = *yes*), left-right ideological identification (1 = *left*; 10 = *right*). On the national level, we also controlled for the quality of life in each country indicated by the HDI score (United Nations Development Programme, 2020). We applied the HDI for every country from the year in which the data was collected in that particular country (2017, 2018, or 2019).

Before our analyses, we had to leave out 3 countries from the EVS dataset, since there was no reported Gini index for the relevant year neither for Azerbaijan nor for Bosnia and Herzegovina, and from Portugal there was no data for one of our key variables, namely for household income. This meant that finally we analyzed data from 31 countries in the EVS dataset (*N* = 51,752),^5^ and 23 countries in the ESS dataset (*N* = 44,387).^6^

### Analytic Strategy

We applied multilevel linear modeling to test our hypotheses, which choice was made due to the nested nature of our dataset, where respondents are grouped according to their nationality. Multilevel modeling is also an appropriate tool to test cross-level interactions, which was one of our goals related to our hypotheses about the contextual influence of income inequality.

When doing the analysis, we set up a random intercept-random slope multilevel model for each database to predict individual-level subjective well-being. In these models, random slopes were assigned to meritocracy belief, since this individual-level predictor was involved in the tested cross-level interaction. The models included Gini index and HDI as country-level predictors, and meritocracy belief, household income, gender, age, education level, subjective health, religiousness, and ideological preference as individual-level predictors. Furthermore, the models included two interaction terms, one between meritocracy belief and income, and a cross-level interaction between meritocracy belief and the Gini index. Individual-level predictors were group-mean centered and country-level predictors were grand-mean centered before the modeling procedure. Our choice of a multilevel modeling approach was supported by the fact that the country-level variance of subjective well-being was significantly different from zero in both cases, what indicates that regardless of between-person differences there are significant differences between countries too, which differences can be explained with country-level predictors (var_*ESS*_ = 0.36; SE = 0.10; *z* = 3.74; *p*<0.001; var_*EVS*_ = 0.26; SE = 0.05; *z* = 5.20; *p*<0.001; ICC_*ESS*_ = 0.109; ICC_*EVS*_ = 0.068).

Parameter estimates were obtained by Bayesian estimation, since there is evidence showing that more accurate parameter estimates can be obtained by it in multilevel models if the number of respondent clusters is limited, what is typically the case with multinational databases like the ESS and the EVS (Hox et al., 2012). Unlike frequentist-based estimations (e.g., maximum likelihood), a Bayesian estimation produces model estimates as distributions of values rather than point estimates. In most cases, the median of this posterior distribution is considered as the most probable model estimate (e.g., coefficient or variance component). Advantages of Bayesian estimation are that it does not rely on any distributional assumptions, and it is able to provide more accurate parameters in the case of small samples, what is the case with international databases with a limited number of countries (Finch and Bolin, 2017).

For the statistical calculations, we used the Mplus 8.5 software (Muthén and Muthén, 1998-2017). When doing the analysis, we used the default non-informative priors of Mplus for the regression coefficients with a normal distribution, a mean of 0, and an infinite variance.^7^ We applied two Markov chain Monte Carlo (MCMC) chains, 400,000 iterations (of which the first half was the burn-in phase), and a thinning rate of 20. This means that the posterior distributions of the model estimates were based on 10,000 iterations (every 20th one after the burn-in phase).

## Results

Descriptive statistics and correlations between the variables are presented in Table 1. The proportion of missing data was low in both databases (ESS: 2.89%; EVS: 3.14%), involved only individual-level variables, was missing at random in both cases (ESS: χ^2^ = 5280.67; *df* = 648; *p* < 0.001; EVS: χ^2^ = 6830.19; *df* = 794; *p* < 0.001), and was handled with multigroup multiple imputation (Enders and Gottschall, 2011) for each country individually before the multilevel modeling.

**TABLE 1:** Descriptive statistics and correlations between variables based on the pooled ESS and EVS datasets.

	1	2	3	4	5	6	7	8	9	10	11
1. HDI	1.00	−0.47***	-0.05***	0.01*	0.01**	0.20***	−0.05***	−0.04***	0.03***	0.11***	0.29***
2. Gini	−0.58***	1.00	0.03***	–0.01	0.03***	−0.07***	0.11***	0.02**	−0.11***	−0.05***	−0.15***
3. Gender	−0.04***	0.02***	1.00	0.04***	0.00	−0.06***	0.15***	−0.04***	−0.09***	−0.06***	–0.01
4. Age	0.09***	−0.04***	0.02**	1.00	−0.08***	−0.40***	0.19***	0.03***	−0.21***	−0.04***	−0.09***
5. Education	0.03***	−0.04***	−0.00	−0.19***	1.00	0.11***	−0.05***	−0.01*	0.24***	0.02***	0.06***
6. Health	0.16***	−0.10***	−0.05***	−0.35***	0.18***	1.00	−0.08***	0.04***	0.25***	0.08***	0.38***
7. Religiousness	−0.15***	0.13***	0.08***	0.08***	−0.06***	−0.03***	1.00	0.13***	−0.10***	–0.00	0.06***
8. Left-right ideology	−0.05***	0.05***	−0.03***	0.02***	−0.05***	0.02***	0.11***	1.00	0.05***	0.16***	0.09***
9. Income	0.16***	−0.12***	−0.10***	−0.20***	0.36***	0.27***	−0.06***	0.03***	1.00	0.08***	0.25***
10. Meritocracy belief	−0.14***	0.12***	−0.03***	−0.02***	0.08***	0.00	0.06***	0.21***	0.06***	1.00	0.09***
11. Subjective well-being	0.21***	−0.18***	−0.00	−0.11***	0.11***	0.46***	0.02***	0.06***	0.26***	0.05***	1.00
ESS—Mean (N)	0.91	30.42	44,387 (23,351 female)	49.14	4.11	3.80	4.50	5.16	5.19	3.02	7.29
ESS—SD	0.04	4.24		18.61	2.93	0.92	3.12	2.24	2.73	1.13	1.83
EVS—Mean (N)	0.88	30.49	51,752 (38,633 female)	49.96	4.24	3.74	51,752 (35,208 religious)	5.45	5.11	5.73	7.10
EVS—SD	0.06	4.43		17.65	1.72	0.94		2.10	2.58	2.86	1.97

*Correlations for the EVS dataset are reported in the bottom triangle, below the diagonal, while correlations for the ESS dataset are reported in the top triangle. ^∗^p < 0.05, ^∗∗^p < 0.01, ^∗∗∗^p < 0.001.*

The Bayesian multilevel model estimations terminated normally with a successful convergence, what is indicated by the fact that the Proportional Scale Reduction (PSR) factor values fell below 1.05 during the first 100 iterations and remained there along the 400,000 iterations in the case of both models. We evaluated the cross-level interaction models based on their *deviance information criterion (DIC)* value against a pair of models without the interaction terms, and another pair of empty models without any predictors. Both in the cases of the ESS and EVS models, the interaction models showed a better fit (DIC_ESS_=165779.11; DIC_EVS_=200320.60) than the models without the interactions (DIC_ESS_=165848.25; DIC_EVS_=200405.83), and the empty models (DIC_ESS_=174382.11; DIC_EVS_=213224.83). This indicates that the predictors and the interaction terms contribute to the models. The details of the interaction models are presented in Table 2.

**TABLE 2:** Multilevel models predicting subjective well-being.

	ESS model				EVS model			
	Est.	*SD*	95% CI	*p*	Est.	*SD*	95% CI	*p*
**Fixed effects**								
Intercept	7.339	0.071	[7.199; 7.480]	<0.001	7.073	0.065	[6.944; 7.204]	<0.001
HDI	14.421	2.301	[9.889; 19.026]	<0.001	4.980	1.398	[2.195; 7.740]	<0.001
Gini	–0.011	0.019	[−0.049; 0.028]	0.278	–0.037	0.017	[−0.072; −0.002]	0.018
Meritocracy belief	0.043	0.014	[0.015; 0.071]	0.003	0.027	0.006	[0.015; 0.039]	<0.001
Income	0.138	0.003	[0.132; 0.145]	<0.001	0.112	0.003	[0.105; 0.118]	<0.001
Gender	0.112	0.015	[0.082; 0.142]	<0.001	0.156	0.015	[0.127; 0.186]	<0.001
Age	0.006	0.000	[0.005; 0.007]	<0.001	0.005	0.000	[0.004; 0.006]	<0.001
Education level	–0.008	0.003	[−0.014; −0.003]	0.001	–0.015	0.005	[−0.024; −0.005]	0.001
Subjective health	0.627	0.009	[0.608; 0.645]	<0.001	0.878	0.009	[0.860; 0.895]	<0.001
Religion	0.054	0.003	[0.049; 0.059]	<0.001	0.197	0.018	[0.162; 0.232]	<0.001
Ideology	0.056	0.004	[0.049; 0.063]	<0.001	0.042	0.004	[0.034; 0.049]	<0.001
Meritocracy belief X Income	–0.015	0.003	[−0.020; −0.009]	<0.001	–0.003	0.001	[−0.005; −0.001]	0.002
Gini X Meritocracy belief	0.007	0.003	[0.000; 0.013]	0.024	0.003	0.001	[0.000; 0.005]	0.031
**Variance of random effects**								
Intercept	0.104	0.044	[0.057; 0.225]	<0.001	0.124	0.040	[0.074; 0.225]	<0.001
Meritocracy belief	0.003	0.002	[0.001; 0.008]	<0.001	0.001	0.000	[0.000; 0.002]	<0.001
**R^2^(Person-level)**	0.177	0.003	[0.170;0.183]	<0.001	0.221	0.003	[0.215;0.227]	<0.001
**R^2^(Country-level)**	0.564	0.109	[0.309;0.731]	<0.001	0.320	0.088	[0.145;0.487]	<0.001

*Reported estimates are the median points of the Bayesian posterior distributions. Est., Unstandardized coefficient; SD, Posterior standard deviation; 95% CI, Upper and lower bounds of the 95% Bayesian credibility interval; P-values indicate the proportion of the posterior distribution that fell to the other side of zero than the median.*

These results showed in the case of both models that HDI was positively related to subjective well-being from the country level predictors (ESS: *b* = 14.42; *p* < 0.001; EVS: *b* = 4.98; *p*<0.001), but the Gini index had a significant negative main effect only in the EVS model (EVS: *b*=−0.04; *p* = 0.018). At the individual-level, all predictors showed a significant relationship with subjective well-being in both models. Being a woman (ESS: *b* = 0.11; *p*<0.001; EVS: *b* = 0.16; *p*<0.001), older (ESS: *b* = 0.01; *p*<0.001; EVS: *b* = 0.01; *p*<0.001), less educated (ESS: *b*=−0.01; *p* = 0.001; EVS: *b*=−0.02; *p* = 0.001), more religious (ESS: *b* = 0.05; *p*<0.001; EVS: *b* = 0.20; *p*<0.001), more right-winger (ESS: *b* = 0.06; *p*<0.001; EVS: *b* = 0.04; *p*<0.001), more wealthy (ESS: *b* = 0.14; *p*<0.001; EVS: *b* = 0.11; *p*<0.001), and healthy (ESS: *b* = 0.63; *p*<0.001; EVS: *b* = 0.88; *p*<0.001), and endorsing meritocracy belief predicted higher subjective well-being (ESS: *b* = 0.04; *p*<0.001; EVS: *b* = 0.03; *p*<0.001). Furthermore, we found an interaction between meritocracy belief and the Gini index on the one hand (ESS: *b* = 0.01; *p* = 0.024; EVS: *b* = 0.003; *p* = 0.031), and income on the other in both models (ESS: *b*=−0.02; p<0.001; EVS: *b*=−0.003; p = 0.002).

To probe the significant two-way interactions further, we ran two separate simple slope analyses (Table 3). The first showed that the strength of relationship between meritocracy belief and subjective well-being gradually increases with the crescendo of the Gini index, and correspondingly the relationship was not significant at a low (−1 SD: *b* = 0.015; *p* = 0.219) and a very low (−2 SD: *b* = −0.013; *p* = 0.334) level of Gini index in the ESS database, and at a very low level of it (−2 SD: *b* = 0.003; *p* = 0.398) in the EVS database. The other simple slope analysis showed that the strength of relationship between meritocracy belief and subjective well-being gradually increases with the drop of income, and correspondingly the relationship was not significant at a high (+ 1 SD: *b* = 0.006; *p* = 0.347) and a very high (+ 2 SD: *b* = −0.031; *p* = 0.058) level of income in the ESS database, and at a very high level of it (+ 2 SD: *b* = 0.011; *p* = 0.089) in the EVS database.

**TABLE 3:** Result of simple slope analyses predicting subjective well-being at different levels of income and Gini indices.

Database	Moderator	Level of the moderator	Est. (*SD*)	95% CI	*P*
ESS	Income	+2 SD	−0.031 (0.020)	[−0.070; 0.008]	0.058
		+1 SD	0.006 (0.016)	[−0.025; 0.037]	0.347
		Mean	0.043 (0.014)	[0.015; 0.071]	0.003
		−1 SD	0.079 (0.016)	[0.048; 0.110]	<0.001
		−2 SD	0.116 (0.019)	[0.077; 0.154]	<0.001
	Gini index	+2 SD	0.098 (0.031)	[0.037; 0.159]	0.002
		+1 SD	0.070 (0.020)	[0.032; 0.109]	<0.001
		Mean	0.043 (0.014)	[0.015; 0.071]	0.003
		−1 SD	0.015 (0.020)	[−0.025; 0.054]	0.219
		−2 SD	−0.013 (0.032)	[−0.076; 0.049]	0.334
EVS	Income	+2 SD	0.011 (0.008)	[−0.005; 0.027]	0.089
		+1 SD	0.019 (0.007)	[0.006; 0.032]	0.004
		Mean	0.027 (0.006)	[0.015; 0.039]	<0.001
		−1 SD	0.034 (0.007)	[0.021; 0.048]	<0.001
		−2 SD	0.042 (0.008)	[0.027; 0.058]	<0.001
	Gini index	+2 SD	0.050 (0.013)	[0.023; 0.076]	<0.001
		+1 SD	0.038 (0.009)	[0.022; 0.055]	<0.001
		Mean	0.027 (0.006)	[0.015; 0.039]	<0.001
		−1 SD	0.015 (0.009)	[−0.002; 0.033]	0.040
		−2 SD	0.003 (0.014)	[−0.023; 0.032]	0.398

*Reported estimates are the median points of the Bayesian posterior distributions. Est., Unstandardized coefficient; SD, Posterior standard deviation; 95% CI, Upper and lower bounds of the 95% Bayesian credibility interval; P-values indicate the proportion of the posterior distribution that fell to the other side of zero than the median.*

In summary, in line with our predictions, we found a positive relationship between meritocracy belief and subjective well-being, and also found that this relationship was stronger on a lower income level (Hypothesis 1), and within the context of higher income inequality (Hypothesis 2). These results could be observed in both models.

## Discussion

Using two recent large-scale international survey databases we found that endorsing meritocracy belief is related to higher level of psychological well-being, which is in line with previous research suggesting that belief in meritocracy is an effective tool to rationalize social inequality (Napier and Jost, 2008; McCoy et al., 2013). It is also worth mentioning that we found this relationship also in the case of prescriptive income meritocracy (measured by the applied item form the EVS). This is important because multiple studies showed so far that it is belief in not prescriptive but descriptive meritocracy which has a system justifying effect (Son Hing et al., 2011; Darnon et al., 2018; Madeira et al., 2019). Nonetheless, in these former studies, prescriptive meritocracy measures neglected the issue of inequality, and mainly concentrated on the principle that rewards and outcomes should (or ought to) bear proportion to people’s actual performance.

In our study, both the prescriptive EVS item and the less univocal ESS item refer to income inequality as a result of the merit principle. Former research showed that both general (Napier and Jost, 2008, Study1) and income egalitarianism (Schneider, 2012) is related to a lower level of subjective well-being, what can explain why even prescriptive meritocracy can have a palliative effect when income inequality, as an inevitable consequence of the merit principle, is explicitly considered. Prescriptive meritocracy belief, when contrasted with prescriptive income egalitarianism (like in the case of the EVS item), can also be related to descriptive meritocracy, since experimental evidence shows that people become more egalitarian if they perceive that merit-based rewarding does not function properly, and tend to tolerate, or even endorse inequality if they see it as an outcome of personal merit (Mitchell and Tetlock, 2009).

We also found that the palliative effect of meritocracy belief was influenced by other factors. Firstly, the results from both databases indicated that the relationship between meritocracy belief and subjective well-being was stronger at a lower personal income level. This is in accordance with the idea that, in terms of subjective well-being, people in a disadvantaged or low-status position have more to benefit from endorsing ideologies that justifies their underdog position in a psychological sense (Osborne and Sibley, 2013; Bahamondes-Correa et al., 2019; Li et al., 2020). Nevertheless, this does not mean that members of low-status groups show a stronger tendency for system justification. It means that the justification of income inequalities promotes the subjective well-being of the poor to a larger extent compared to those better-off. As a matter of fact, there is evidence showing that members of high-status groups tend to justify inequality more strongly than members of low-status groups (Owuamalam et al., 2019). For them, it might be the material and other rewards gained from the unequal system that elevates happiness and satisfaction, whereas for the poor, the mere perception of their own position as just and deserved seem to have a direct effect on subjective well-being, at least in the short run. Furthermore, promoting ideologies of meritocracy not only justifies the status of those on the top of the social hierarchies, but through its increased palliative effects for low status people, it keeps them satisfied, therefore, unlikely to protest against structural inequalities.

An interesting question is why and how meritocracy belief contributes to the short-term well-being of the poor, which question should be addressed by further investigation in the future. One potential mediator can be one’s belief in social mobility. It is already known that belief in social mobility is related to system justification (Jost and Hunyady, 2005; Day and Fiske, 2017; Li et al., 2020), and subjective well-being (Li et al., 2020). It is possible that this belief is especially relieving to those in a disadvantageous position. If they think that meritocracy works properly, they can assume that it is possible to advance their social position by hard work and personal efforts, which assumption can promote psychological well-being.

We also found in both datasets that the relationship between (income) meritocracy belief and subjective well-being is stronger in countries characterized by a high (vs. low) level of income inequality. It seems that countries with high level in inequalities are more vulnerable to social unrest and consequently more dependent on justifying ideologies to maintain their social structure through the palliative effects of meritocracy belief. This finding is in line with previous results showing that the palliative effect of different justifying ideologies like conservatism (Napier and Jost, 2008), symbolic racism (Sengupta et al., 2017), or sexism (Napier et al., 2010) is larger in more unequal contexts.

Our findings contradict studies which found that members of high- and low-status groups benefit equally from the palliative effect of justifying ideologies (Napier et al., 2010; Sengupta et al., 2017; Vargas-Salfate et al., 2018). This may be because we investigated the effect of income as a hierarchy-establishing factor, whereas Napier et al. (2010) tested for gender inequality, and Sengupta et al. (2017) investigated ethnic relations. It is possible that people benefit differently from the justification of inequality within different hierarchy systems. Since the idea of ethnic and gender equality is a strong cultural norm in most Western countries, rationalizing such inequalities might have a palliative benefit for everyone independently from group membership. In contrast with such kind of inequalities, the topic of income inequatilty (vs. equality and governmental redistribution) is still a dividing ideological topic. Consequently, rationalizing a disadvantageous status within this particular hierarchy might result in a stronger palliative benefit for the poor.

Our results also contradict Vargas-Salfate et al. (2018) multinational study, in which neither country-level income inequality nor subjective social status influenced the relationship between system-justification and subjective well-being. Nonetheless, in their study, contextual inequality was based on income, while the potential justifying belief was general system justification, so there was no direct conceptual correspondence between the investigated social hierarchy and the justifying ideology. We cannot rule out the possibility that people approve (or disapprove) of the system in general regardless of the scale of income differences in their own country or their own subjective economic position. Or to put it in another way, when answering the items of the system justification scale (Kay and Jost, 2003), it is possible that respondents’ answers were based on other more salient factors, but not on the extent of income inequalities. In our study, we tested the palliative function of meritocracy belief with items directly referring to the question of financial equality to ensure a conceptual correspondence of the social hierarchy and the justifying ideology (see also Sengupta et al., 2015; Bahamondes-Correa et al., 2019).

### Limitations

Although working with two international databases allowed us to work with data collected in 34 countries in total and on robust samples, both databases consist mainly of data collected in European countries, that makes the cultural generalizability of our results somewhat limited. Further research should test the interplay between income inequality and the palliative effect of meritocracy belief with more diverse international data.

Additionally, we were constrained by the operationalizations in these surveys, and consequently, most of the analyzed variables were measured by single items instead of validated multiple-item scales. In line with this, our subjective well-being variable was based on only two items, self-reported happiness and life satisfaction, which are often considered as an evaluative or cognitive aspect of subjective well-being representing only one dimension of the well-being concept (e.g., Steptoe et al., 2015; Charalampi et al., 2018). Other studies investigated the palliative effect of different ideological beliefs with other subjective well-being indicators, like physical health or negative emotional responses (e.g., Bahamondes-Correa, 2016; Vargas-Salfate et al., 2018; Suppes et al., 2019). Another related limitation is that we could not strictly and univocally differentiate between descriptive and prescriptive meritocracy belief in terms of measurement in the case of the ESS database, because the item could be interpreted in both ways. This prevented us from making systematic comparisons between the palliative function of prescriptive and descriptive meritocracy belief, what is an important task to be done in the future.

Relying on the cross-sectional design of these datasets did not allow us to draw causal conclusions between subjective well-being and the acceptance of meritocracy. Whereas theoretically it is possible that happy and satisfied people tend to accept meritocracy belief and other system-justifying ideologies more rather than system-justification enhancing psychological well-being, previous evidence from experimental and longitudinal studies support the idea that acceptance (or salience) of different sorts of system-justifying beliefs indeed leads to a higher level of subjective well-being (e.g., Wakslak et al., 2007; Vargas-Salfate et al., 2018). Besides, as it was mentioned earlier, the palliative function of ideological rationalization seems to work only in the short run, but its long-term relationship with subjective well-being turns to be negative after a while (Harding and Sibley, 2013; Godfrey et al., 2019). For these reasons, future research should also focus on and reveal the accurate temporal dynamics between meritocracy belief and subjective well-being.

## Conclusion

The results of our study involving almost 100,000 respondents from 36 countries suggested that both micro- and macro-level income inequalities influence the relationship between meritocracy belief and subjective well-being. This indicates that the direct psychological pay-off of rationalizing the *status quo* can be further enhanced by contextual factors like one’s disadvantaged social status or the observable scale of inequality of a country. One of the most important implications of this tendency is that the short-term emotional relief achieved by ideologies that serve to justify the *status quo*, such as meritocracy belief, might prevent social change efforts toward material equality by those who would be the primary beneficiaries of such a change, namely the poor, especially in highly unequal contexts where such change would be most needed. In summary, ideologies, such as the “American Dream”—paradoxically an idea that was supposed to promote equal opportunities—can prevent social change efforts to reduce inequalities in two ways: by offering justification for privileges of high status people and by keeping low status individuals happily in “their place” through its palliative effect.

## Data Availability Statement

The original contributions presented in the study are included in the article/Supplementary Material, further inquiries can be directed to the corresponding author/s.

## Author Contributions

MH organized the database, performed the statistical analyses, and wrote the first draft of the manuscript. All authors contributed to manuscript revision, read, and approved the submitted version, contributed to conception and design of the study.

## Conflict of Interest

The authors declare that the research was conducted in the absence of any commercial or financial relationships that could be construed as a potential conflict of interest.

## Publisher’s Note

All claims expressed in this article are solely those of the authors and do not necessarily represent those of their affiliated organizations, or those of the publisher, the editors and the reviewers. Any product that may be evaluated in this article, or claim that may be made by its manufacturer, is not guaranteed or endorsed by the publisher.

## Footnotes

1 We also tested whether the combination of the two moderating factors (i.e., high and low personal income within high and low country level income differences) would predict different palliative effects of meritocracy belief. Namely, we tested whether the pressure on those with a lower (vs. higher) income is larger to justify their disadvantaged position within more unequal (vs. equal) contexts. We tested this three-way interaction as an exploratory hypothesis in the absence of previous evidence suggesting a specific direction of the effect. The only study investigating a similar question found that the palliative benefits of accepting symbolic prejudice as a system-justifying ideology were distributed equally among ethnic minority and majority New Zealanders, and this effect was not moderated by regional inequality (Sengupta et al., 2017). Therefore, in this study the palliative effect moderated by contextual inequality was not further affected by social position. Our exploratory hypothesis was tested in the same manner (based on the same datasets) as the other hypotheses, but the assumed three-way interaction turned out to be non-significant in the relevant models, which are reported in Supplementary Table 2.

2
https://www.europeansocialsurvey.org/docs/round8/questionnaire/ESS8_welfare_final_module_template.pdf

3 For more information on the ESS income measure: https://www.europeansocialsurvey.org/docs/round8/survey/ESS8_appendix_a2_e02_1.pdf

4
https://europeanvaluesstudy.eu/methodology-data-documentation/survey-2017/pre-release-evs-2017/documentation-survey-2017/

5 Albania, Austria, Armenia, Bulgaria, Belarus, Croatia, Czechia, Denmark, Estonia, Finland, France, Georgia, Germany, Hungary, Iceland, Italy, Lithuania, Montenegro, Netherlands, Norway, Poland, Romania, Russia, Serbia, Slovakia, Slovenia, Spain, Sweden, Switzerland, North Macedonia, Great Britain. For more information on the sampling and data collection procedure of the European Values Study: https://europeanvaluesstudy.eu/methodology-data-documentation/survey-2017/pre-release-evs-2017/documentation-survey-2017/

6 Austria, Belgium, Czech Republic, Estonia, Finland, France, Germany, Hungary, Iceland, Ireland, Israel, Italy, Lithuania, Netherlands, Norway, Poland, Portugal, Russian Federation, Slovenia, Spain, Sweden, Switzerland, United Kingdom. For more information on the sampling and data collection procedure of the European Social Survey: https://www.europeansocialsurvey.org/methodology/ess_methodology/sampling.html https://www.europeansocialsurvey.org/methodology/ess_methodology/data_collection.html

7 To make sure that these non-informative priors did not affect substantially the final results, each reported model was rerun with somewhat different weakly informative priors for the coefficients. These priors were based on the results of the same models with maximum likelihood estimation, had a normal distribution, zero as mean, and a variance corresponding to the standard errors of the ML estimation, as suggested by van Zwet (2019). The models with these alternative priors are reported in Supplementary Table 1. These models show basically the same results as the ones with the default priors, what suggests that our results were not affected by the chosen priors.
