# Supplementary material for: The Relationship Between Income Inequality and the Palliative Function of Meritocracy Belief: The Micro- and the Macro-Levels Both Count
Source: Front Psychol. 2021 Oct 8;12:709080. doi: 10.3389/fpsyg.2021.709080 (PMC8531093; doi:10.3389/fpsyg.2021.709080)
Supplement: Supplementary file 1 [file Table_1.DOCX]

**Online Supplementary Material**

| Table 1 | | | | | | | | |
| --- | --- | --- | --- | --- | --- | --- | --- | --- |
| Multilevel models predicting subjective well-being with alternative weakly informative priors. | | | | | | | | |
|  | ESS Model | | | | EVS Model | | | |
|  | *Est.* | *SD* | *95% CI* | *p* | *Est.* | *SD* | *95% CI* | *p* |
| **Fixed effects** |  |  |  |  |  |  |  |  |
| Intercept | 7.339 | 0.069 | [7.202; 7.477] | < .001 | 7.073 | 0.065 | [6.946; 7.202] | < .001 |
| HDI | 14.141 | 1.187 | [11.815; 16.456] | < .001 | 5.005 | 0.916 | [3.205; 6.808] | < .001 |
| Gini | -0.012 | 0.017 | [-0.046; 0.022] | .235 | -0.037 | 0.016 | [-0.067; -0.006] | .010 |
| Meritocracy belief | 0.042 | 0.014 | [0.014; 0.070] | .003 | 0.027 | 0.006 | [0.014; 0.039] | < .001 |
| Income | 0.138 | 0.003 | [0.132; 0.145] | < .001 | 0.112 | 0.003 | [0.105; 0.118] | < .001 |
| Gender | 0.111 | 0.015 | [0.081; 0.140] | < .001 | 0.155 | 0.015 | [0.126; 0.185] | < .001 |
| Age | 0.006 | 0.000 | [0.005; 0.007] | < .001 | 0.005 | 0.000 | [0.004; 0.006] | < .001 |
| Education level | -0.008 | 0.003 | [-0.014; -0.003] | .001 | -0.014 | 0.005 | [-0.024; -0.005] | .001 |
| Subjective health | 0.624 | 0.009 | [0.605; 0.642] | < .001 | 0.875 | 0.009 | [0.858; 0.893] | < .001 |
| Religion | 0.054 | 0.003 | [0.049; 0.059] | < .001 | 0.195 | 0.018 | [0.160; 0.230] | < .001 |
| Ideology | 0.056 | 0.004 | [0.049; 0.063] | < .001 | 0.042 | 0.004 | [0.034; 0.049] | < .001 |
| Meritocracy belief X Income | -0.015 | 0.003 | [-0.020; -0.009] | < .001 | -0.003 | 0.001 | [-0.005; -0.001] | .002 |
| Gini X Meritocracy belief | 0.007 | 0.003 | [0.000; 0.013] | .024 | 0.003 | 0.001 | [0.000; 0.005] | .031 |
| **Variance of random effects** |  |  |  |  |  |  |  |  |
| Intercept | 0.100 | 0.041 | [0.055; 0.211] | < .001 | 0.121 | 0.038 | [0.073; 0.218] | < .001 |
| Meritocracy belief | 0.003 | 0.002 | [0.001; 0.008] | < .001 | 0.001 | 0.000 | [0.000; 0.002] | < .001 |
| **R^2^ (Person-level)** | .176 | .003 | [.170; .182] | < .001 | .220 | .003 | [.214; .226] | < .001 |
| **R^2^ (Country-level)** | .561 | .090 | [.360; .712] | < .001 | .318 | .079 | [.164; .471] | < .001 |
| *Note.* Reported estimates are the median points of the Bayesian posterior distributions. Est. = Unstandardized coefficient; SD = Posterior standard deviation; 95% CI = Upper and lower bounds of the 95% Bayesian credibility interval; *P*-values indicate the proportion of the posterior distribution that fell to the other side of zero than the median. | | | | | | | | |

| Table 2 | | | | | | | | |
| --- | --- | --- | --- | --- | --- | --- | --- | --- |
| Multilevel models predicting subjective well-being with a three-way interaction between the Gini index, income, and meritocracy belief. | | | | | | | | |
|  | ESS Model | | | | EVS Model | | | |
|  | *Est.* | *SD* | *95% CI* | *p* | *Est.* | *SD* | *95% CI* | *p* |
| **Fixed effects** |  |  |  |  |  |  |  |  |
| Intercept | 7.340 | 0.070 | [7.204; 7.480] | < .001 | 7.073 | 0.065 | [6.945; 7.203] | < .001 |
| HDI | 14.370 | 2.289 | [9.867; 18.980] | < .001 | 4.941 | 1.399 | [2.223; 7.730] | < .001 |
| Gini | -0.011 | 0.019 | [-0.050; 0.027] | .272 | -0.038 | 0.017 | [-0.072; -0.004] | .016 |
| Meritocracy belief | 0.046 | 0.013 | [0.021; 0.071] | < .001 | 0.027 | 0.006 | [0.015; 0.039] | < .001 |
| Income | 0.140 | 0.015 | [0.111; 0.169] | < .001 | 0.115 | 0.007 | [0.101; 0.129] | < .001 |
| Gender | 0.117 | 0.015 | [0.087; 0.147] | < .001 | 0.157 | 0.015 | [0.127; 0.187] | < .001 |
| Age | 0.006 | 0.000 | [0.005; 0.007] | < .001 | 0.005 | 0.000 | [0.005; 0.006] | < .001 |
| Education level | -0.008 | 0.003 | [-0.014; -0.003] | .001 | -0.015 | 0.005 | [-0.024; -0.005] | .001 |
| Subjective health | 0.624 | 0.009 | [0.605; 0.642] | < .001 | 0.874 | 0.009 | [0.857; 0.892] | < .001 |
| Religion | 0.054 | 0.003 | [0.049; 0.059] | < .001 | 0.196 | 0.018 | [0.161; 0.231] | < .001 |
| Ideology | 0.058 | 0.004 | [0.050; 0.065] | < .001 | 0.042 | 0.004 | [0.035; 0.050] | < .001 |
| Meritocracy belief X Income | -0.015 | 0.005 | [-0.025; -0.005] | .004 | -0.004 | 0.002 | [-0.009; -0.000] | .025 |
| Gini X Meritocracy belief | 0.007 | 0.003 | [0.001; 0.013] | .016 | 0.002 | 0.001 | [0.000; 0.005] | .035 |
| Income X Gini | 0.000 | 0.003 | [-0.007; 0.007] | .447 | 0.001 | 0.002 | [-0.002; 0.004] | .306 |
| Gini X Income X Meritocracy belief | 0.001 | 0.001 | [-0.002; 0.004] | .201 | 0.000 | 0.000 | [-0.001; 0.001] | .164 |
| **Variance of random effects** |  |  |  |  |  |  |  |  |
| Intercept | 0.105 | 0.043 | [0.057; 0.221] | < .001 | 0.123 | 0.040 | [0.073; 0.224] | < .001 |
| Meritocracy belief | 0.002 | 0.001 | [0.001; 0.006] | < .001 | 0.001 | 0.000 | [0.000; 0.002] | < .001 |
| Income | 0.004 | 0.002 | [0.002; 0.009] | < .001 | 0.001 | 0.000 | [0.001; 0.002] | < .001 |
| Meritocracy belief X Income | 0.000 | 0.000 | [0.000; 0.001] | < .001 | 0.000 | 0.000 | [0.000; 0.000] | < .001 |
| **R^2^ (Person-level)** | .182 | .003 | [.177; .189] | < .001 | .224 | .003 | [.218; .230] | < .001 |
| **R^2^ (Country-level)** | .559 | .107 | [.315; .726] | < .001 | .319 | .088 | [.146; .489] | < .001 |
| *Note.* Reported estimates are the median points of the Bayesian posterior distributions. Est. = Unstandardized coefficient; SD = Posterior standard deviation; 95% CI = Upper and lower bounds of the 95% Bayesian credibility interval; *P*-values indicate the proportion of the posterior distribution that fell to the other side of zero than the median. | | | | | | | | |

**Syntax for the interaction models (MPlus)**

Title: ess;

Data: file is essdata.dat;

Variable:

names are

country swb hdi gini merit income gender age

edu health religion ideology;

usevariables are

country swb hdi gini merit income gender age

edu health religion ideology; iXm;

within are

gender age edu health religion

ideology income merit iXm;

between are

hdi gini;

cluster = country;

define:

center hdi (grandmean);

center gini (grandmean);

center merit (groupmean);

center gender (groupmean);

center age (groupmean);

center edu (groupmean);

center health (groupmean);

center income (groupmean);

center ideology (groupmean);

center religion (groupmean);

iXm=income*merit;

Analysis:

type = twolevel random;

estimator=bayes;

fbiterations=20000;

thin=20;

Model:

%within%

swb on gender age edu health ideology religion

income iXm;

merit_s | swb on merit;

%between%

swb on hdi gini;

merit_s on gini;
